# Investigating the effect of anatomical variations in the response of the neonatal brachial plexus to applied force: Use of a two-dimensional finite element model

**DOI:** 10.1371/journal.pone.0303511

**Published:** 2024-05-14

**Authors:** Sarah J. Wright, Michele J. Grimm

**Affiliations:** 1 Department of Biomedical Engineering, Michigan State University, East Lansing, MI, United States of America; 2 Department of Mechanical Engineering, Michigan State University, East Lansing, MI, United States of America; 3 College of Nanotechnology, Science, and Engineering, University at Albany, Albany, NY, United States of America; AIIMS: All India Institute of Medical Sciences, INDIA

## Abstract

The brachial plexus is a set of nerves that innervate the upper extremity and may become injured during the birthing process through an injury known as Neonatal Brachial Plexus Palsy. Studying the mechanisms of these injuries on infant cadavers is challenging due to the justifiable sensitivity surrounding testing. Thus, these specimens are generally unavailable to be used to investigate variations in brachial plexus injury mechanisms. Finite Element Models are an alternative way to investigate the response of the neonatal brachial plexus to loading. Finite Element Models allow a virtual representation of the neonatal brachial plexus to be developed and analyzed with dimensions and mechanical properties determined from experimental studies. Using ABAQUS software, a two-dimensional brachial plexus model was created to analyze how stresses and strains develop within the brachial plexus. The main objectives of this study were (1) to develop a model of the brachial plexus and validate it against previous literature, and (2) to analyze the effect of stress on the nerve roots based on variations in the angles between the nerve roots and the spinal cord. The predicted stress for C5 and C6 was calculated as 0.246 MPa and 0.250 MPa, respectively. C5 and C6 nerve roots experience the highest stress and the largest displacement in comparison to the lower nerve roots, which correlates with clinical patterns of injury. Even small (+/- 3 and 6 degrees) variations in nerve root angle significantly impacted the stress at the proximal nerve root. This model is the first step towards developing a complete three-dimensional model of the neonatal brachial plexus to provide the opportunity to more accurately assess the effect of the birth process on the stretch within the brachial plexus and the impact of biological variations in structure and properties on the risk of Neonatal Brachial Plexus Palsy.

## Introduction

Neonatal brachial plexus palsy (NBPP) occurs during the birthing process in approximately 1.5/1,000 total births [1]. When the injury persists past 12 months of age, continuing outcomes may include joint subluxation, muscle weakness, and sensory dysfunction [2]. Until the early 1990’s, it was thought that NBPP solely occurred due to trauma induced by lateral neck traction caused by the birthing attendant (exogenous force). Since then, clinical evidence has demonstrated that both temporary and permanent NBPP can occur due to other mechanisms of injury. Factors affecting the mechanism of injury may include bending of the neck, fetal malpositioning, labor induction, labor abnormalities, operative vaginal delivery, and shoulder dystocia [1].

NBPP injuries are difficult to research clinically due to ethical issues that surround the vulnerable subject group—infants. Finite element models (FEM) present an opportunity to investigate various aspects of NBPP. Currently, there is no FEM model in the literature on the neonatal brachial plexus and spinal cord. The objective of this study was to create a two-dimensional model of the neonatal brachial plexus that could be used to investigate how stress develops in the various brachial plexus roots when loads and displacements are applied. The first phase of the project focuses on the development and validation of the model. The second phase focuses on analyzing the effect of stress on the nerve roots based on variations in the angles between the nerve roots and the spinal cord.

## Methods

**Fig 1:**
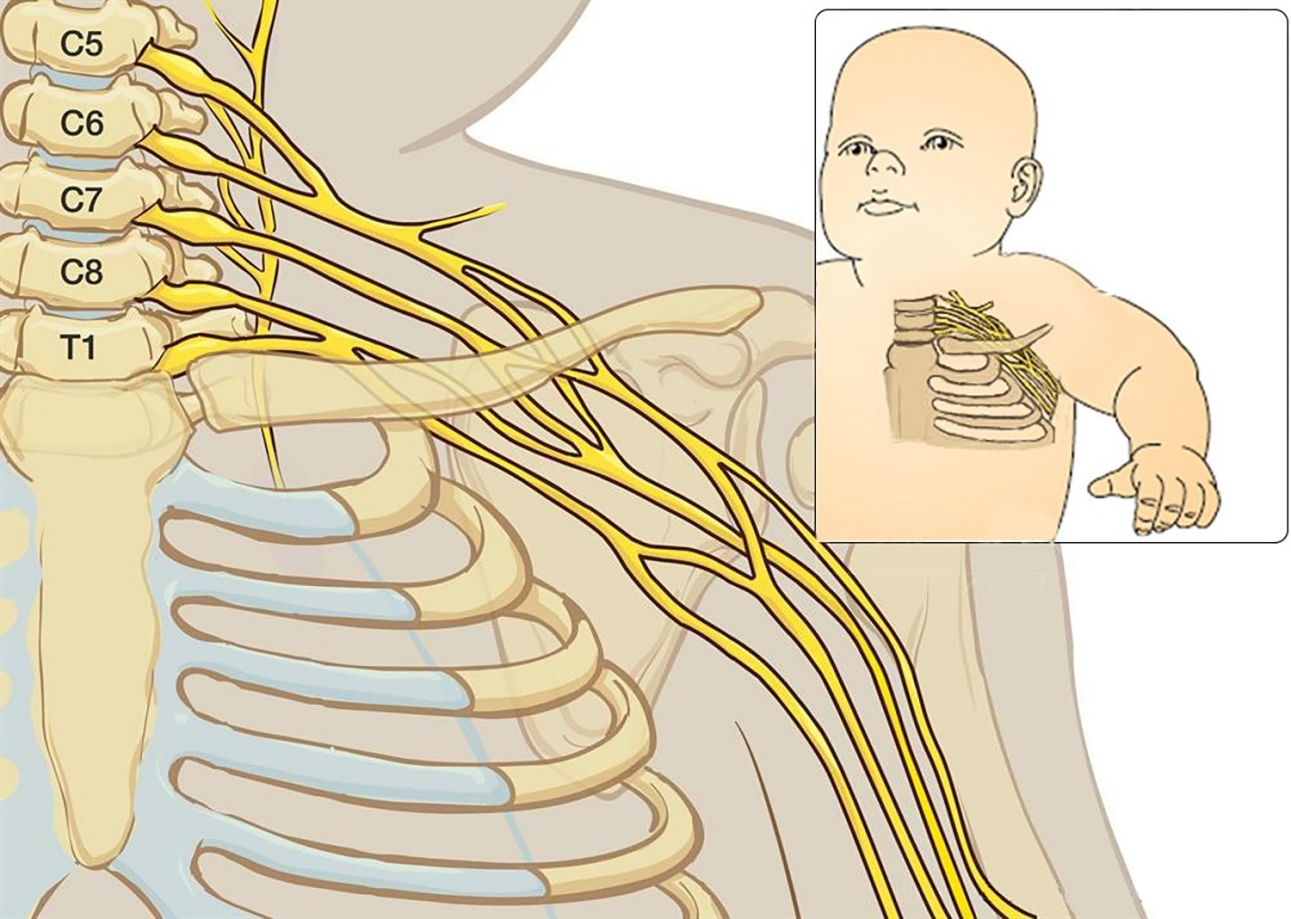
Anatomy of the brachial plexus. The brachial plexus is made of five sections: Roots, trunks, divisions, cords, and terminal branches (© 2004–2023 AboutKidsHealth)[3].

ABAQUS CAE (v. 2022, Dassault Systèmes) was used to design, mesh, and analyze a brachial plexus and spinal cord model of a human neonate. Vertebral bodies and connective tissue surrounding the spinal cord and its connection to the nerve roots were not included. While in reality the brachial plexus is divided into five sections–roots, trunks, divisions, cords, and terminal nerves, our 2D model was constructed solely of the roots and trunks due to the 3D, anatomical complexity that occurs within the division section (Fig 1).

The model was designed based on dimensions measured during primary reconstructive surgery. These dimensions are provided in Table 1 and include the length of the sections as well as both the transverse and cranial-caudal dimensions of each nerve root. The cranial-caudal dimensions were used to represent the thickness of the nerves in this 2D model, as that dimension falls within the plane of analysis. The original anatomy was collected by the University of Michigan’s Neurosurgery department and averaged based on measurements made from the brachial plexus’ roots and trunks of 23 human infants.

**Table 1:** Average dimensions for infant brachial plexus.

(mm)	C5	C6	C7	C8	T1
**Cranial Caudal Root Diameter**	3	3.1	3.8	2.7	2.5
**Transverse Root Diameter**	1.1	1.3	1.6	1.4	1.4
**Spinal root to dorsal root ganglion**	7.5	7.5	7.5	7.5	7.5
**Dorsal root ganglion to end of bony foramen**	9	8	7	5.5	-
**Foramen to formation of trunk**	14	15.5	19	7	6

Two cases were developed to represent the difference between quasistatic stretch (0.01 mm/second) and dynamic stretch (10 mm/second) of the nerves, as seen in Table 2. Within ABAQUS software, boundary conditions and loads were established. The boundary conditions were the same for both cases, including an encastré condition to the midline of the spinal cord and XYSMM conditions to the superior and inferior portions of the five nerves. The encastré boundary condition constrained all active structural degrees of freedom within the edge selected and the XYSMM boundary condition allowed the nerve to stretch only in the plane of the model when loads were applied.

**Table 2:** Mechanical properties of the neonatal brachial plexus and spinal cord–measured in piglet brachial plexus roots at two loading rates [4,5].

Case	Loading Rate [mm/sec]	Maximum Load [N]	Young’s Modulus [MPa]	Poisson’s Ratio	Applied Pressure at Distal Trunk [MPa]
**1**	0.01	1.08	1.48	0.4	0.332
**2**	10	2.12	2.02	0.4	0.653

To date, there has been no assessment of the mechanical properties of the human neonatal brachial plexus. Thus, the Young’s modulus and Poisson’s ratio were from data collected from neonatal piglets, as seen in Table 2 [4,5]. The same material properties were used for both the brachial plexus and the spinal cord, as the model was constructed as a single, deformable structure.

In phase one, a load was applied perpendicular to the plane of the distal end of each trunk, and the resulting stress was evaluated against *in vitro* tensile tests conducted on neonatal piglets. In phase two, the model was changed to evaluate the effect of different nerve root angles on the stress that develops within the nerve root. Loads were again applied perpendicular to the distal end of each trunk. In both phases of the study, the values of stress were analyzed by identifying the element with the maximum stress at the junction between the nerve root and the spinal cord.

### Phase one–Validation of two-dimensional model

Two different loading conditions were examined with the model, as summarized in Table 2. Both simulated a pulling force applied to the nerves at the distal end of the trunk, as would occur with the depression of the shoulder while the head and neck remained aligned with the axis of the spine. Two different loading rates were used, to match experimentally available data for validation–and these affected both the Young’s modulus and the maximum load applied, which increase at higher loading rates due to the viscoelastic nature of nerves. The applied load values were selected to match the experimental study of Dr. Singh [5].

A tensile force of 5x the load to rupture a single nerve root (Table 2) was divided between the trunks based on composite theory. As the Young’s modulus was constant for the three trunk levels, the load to each trunk was proportional to its cross-sectional area. The stretch-inducing load was modeled as an applied pressure distributed over the distal end of each nerve trunk–at the junction with the division. Thus, the upper trunk connected to the C5 and C6 nerve roots, the middle trunk was the extension of the C7 nerve root, and the lower trunk included the C8 and T1 nerve roots. The magnitude of the pressure was calculated based on the share of the load on each trunk normalized by the estimated cross-sectional area. The cross-sectional area of each nerve root was calculated based on an ellipse with the major and minor axes equal to the cranial-caudal and transverse dimensions (Table 1), and the areas used were then summed for the upper (C5 + C6) and lower (C8 + T1) trunks, assuming that the tissue volume (and thus cross-sectional area) was maintained as the roots combined into trunks. As area factored into both the distribution of the force across the five nerve roots and the conversion of the force on each trunk to an applied pressure, the pressure was equal for each of the trunks (Table 2). The applied pressure for the distal portions of the three trunks in Case 1 was 0.332 MPa. For Case 2, the applied pressure was 0.653 MPa.

### Phase two–Anatomical variations

**Fig 2:**
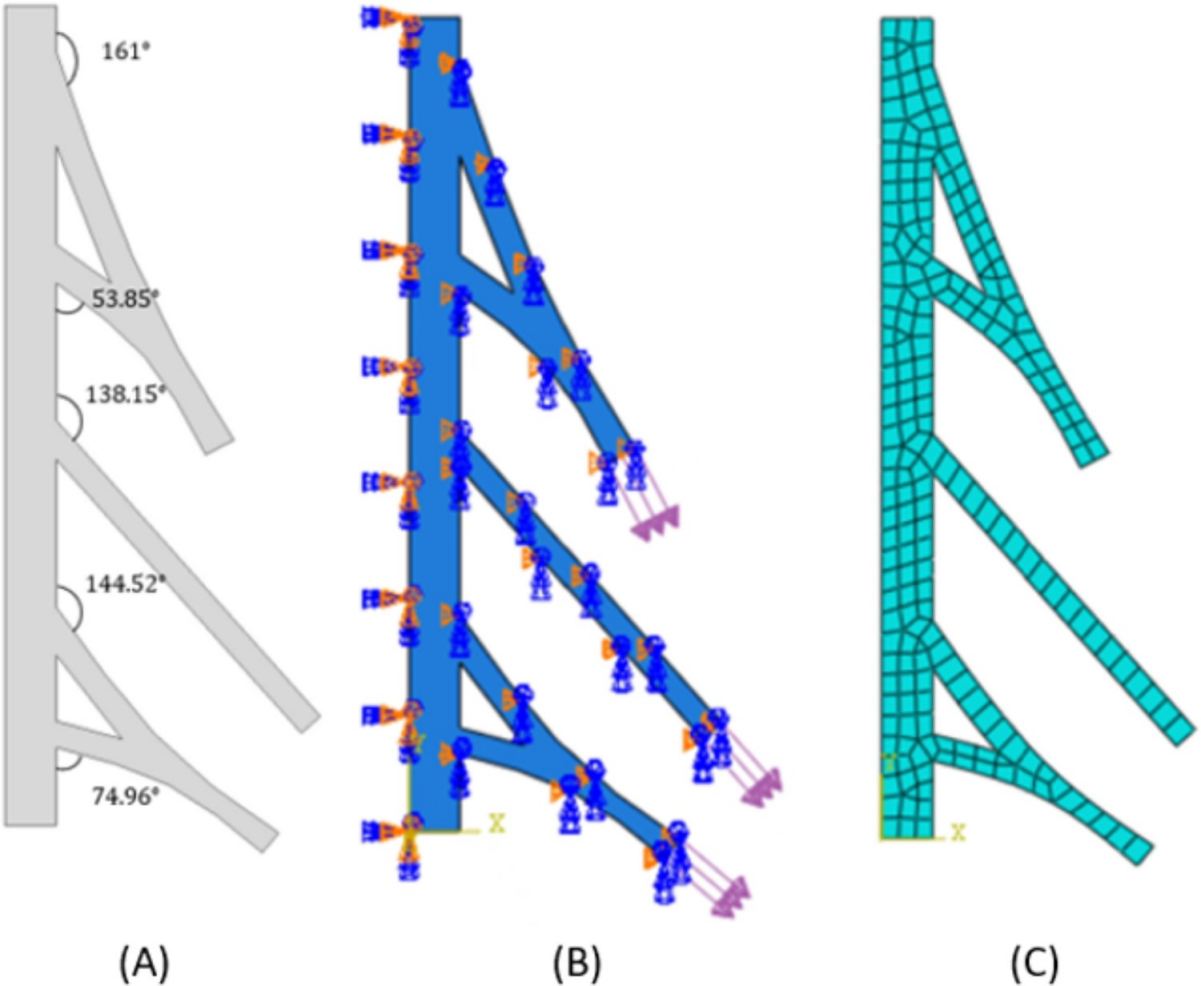
(A) Original geometry of the spinal cord + brachial plexus root/trunk model with the initial angles at each nerve root indicated; (B) Boundary conditions and stretch-inducing pressure applied to distal ends; and (C) Mesh of 2D FEM of a neonatal brachial plexus and spinal cord.

In phase one, a two-dimensional model of a neonatal brachial plexus was created and validated in comparison to an *in vitro* neonatal piglet study (see Results). The angles were constrained for C5/C6 and C7/C8 based on the clinically-measured lengths of the respective nerve roots (Table 1). This base-line geometry was reviewed by the University of Michigan Neonatal Brachial Plexus Program team to confirm that it was reasonable compared to *in vivo* observation. Phase two used the validated model and created anatomical variations in order to evaluate the effect of different nerve root angles on the stress that develops within the nerve root. The starting angle for each nerve root was measured in ABAQUS for phase one—the validated model (Fig 2). The anatomy of the validated model was then adjusted to represent a change in the angle between each nerve root and the spinal cord (± three and six degrees), the same stretch-inducing pressures were applied to the distal end of the trunks, and the stress in the proximal nerve root was then evaluated.

## Results

### Phase one–Validation of two-dimensional model

A mesh convergence was conducted to determine the number of required elements to ensure that the results of the analysis are not affected by the mesh size and therefore provide an accurate solution. A maximum global mesh size of 2 mm and a minimum mesh size of 0.2 mm was used throughout the model. The model was meshed with a total of 304 nodes and 212 elements– 206 linear quadrilateral (CPS4R) and 6 triangular (CPS4). Table 3 provides the maximum values of stress predicted at each nerve root by the model for both loading cases. All stress measurements were taken at the proximal end of the nerve root, where it intersects with the spinal cord.

**Table 3:** Maximum stress values in the proximal nerve root for Case 1 (low loading-rate properties– 0.01 mm/sec) and Case 2 (high loading-rate properties– 10 mm/sec). Stress values were identified at the proximal end of the nerve root, where it joins with the spinal cord.

Von Mises Stress [MPa]	Comparison Maximum Nerve Trunk/Root Stress–Experimental Mean [5]	C5	C6	C7	C8	T1
**Case 1**	0.200	0.246	0.250	0.236	0.171	0.181
**Case 2**	0.450	0.486	0.495	0.466	0.327	0.357

Our model was validated through comparison with the results of Dr. Anita Singh [5]. Dr. Singh calculated a stress in the piglet brachial plexus corresponding to the experimentally-measured failure load. For Case 1, the maximum stress found experimentally was 0.200 MPa, and for Case 2 it was 0.450 MPa. In our model, the predicted maximum Von Mises stress for C5 in Case 1 was 0.246 MPa, and in Case 2 was 0.486 MPa (Table 3). C5 was selected as the nerve root for comparison in this validation because it is the first nerve root that experiences failure in a brachial plexus injury when the arm is adducted. C5 and C6 nerve roots experience the highest stress and the largest displacement in comparison to the lower nerve roots, which correlates with clinical patterns of injury.

**Fig 3:**
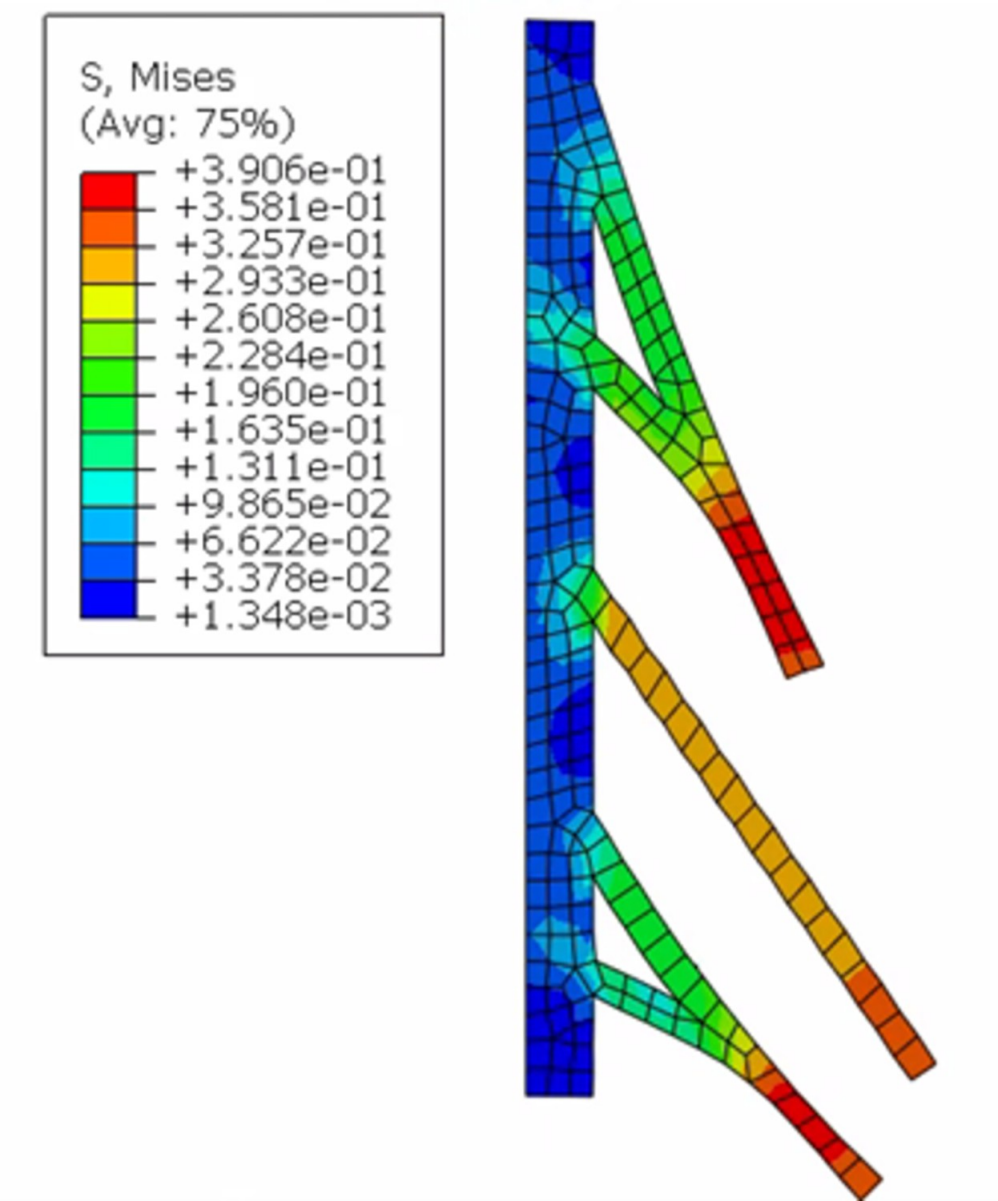
Von mises stress results of Case 1 model (low loading-rate properties– 0.01 mm/sec).

The results are presented visually for Case 1 in Fig 3. The pattern of stresses was the same for Case 1 and Case 2, as the nerve roots were modeled as homogeneous, linear elastic materials. The alignment between the predicted stress at the proximal nerve root of C5 and the experimentally measured failure stress for an isolated root/trunk segment gave confidence that the model was reasonably biofidelic and that it could be used for other parametric analyses.

### Phase two–Anatomical variations

**Fig 4:**
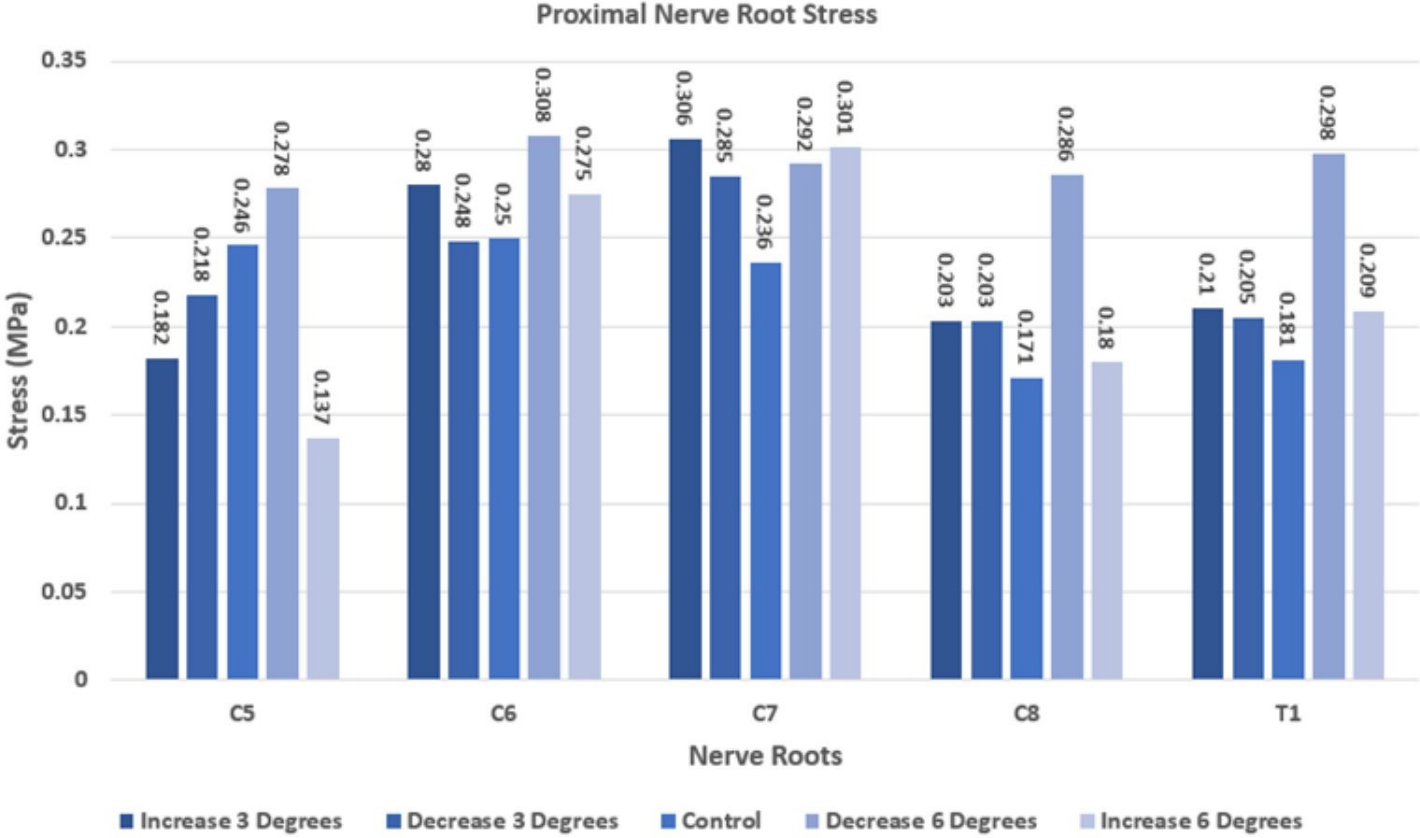
Results of Case 1 model (low loading-rate properties– 0.01 mm/sec) with a range of nerve root angle iterations.

Using the validated model, variations from the original anatomy in the angle between each nerve root and the spinal cord (± three and six degrees) were created, and the stress in the proximal nerve root was then evaluated. The variation in predicted Von Mises stress is reported in Table 4 and Fig 4.

**Table 4:** Results of Case 1 model (low loading-rate properties– 0.01 mm/sec) with a range of nerve root angle iterations.

	Maximum Stress Values at the Nerve Root [MPa and %]				
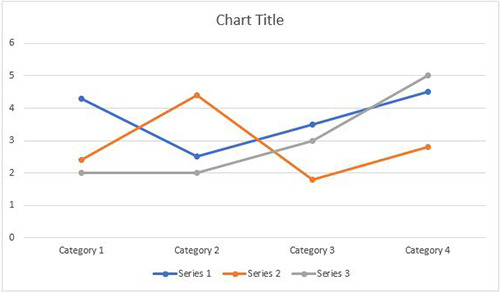	6 Degree Increase	3 Degree Increase	Original Anatomy	3 Degree Decrease	6 Degree Decrease
**C5**	0.137 (-44%)	0.182 (-26%)	0.246	0.218 (-11%)	0.278 (+13%)
**C6**	0.275 (+10%)	0.280 (+12%)	0.250	0.248 (-1%)	0.308 (+23%)
**C7**	0.301 (+28%)	0.306 (+30%)	0.236	0.285 (+21%)	0.292 (+24%)
**C8**	0.180 (+5%)	0.203 (+19%)	0.171	0.203 (+19%)	0.286 (+67%)
**T1**	0.209 (+15%)	0.210 (+16%)	0.181	0.205 (+13%)	0.298 (+65%)

There was no clear pattern in the change in stress predicted at the junction of the nerve root and spinal cord for the variations in nerve root angle. At C5, the stress decreased to 56% of the original baseline value when the angle between the nerve root and the spinal cord increased by only 6 degrees. With a 6 degree decrease in angle with respect to the spinal cord, the model predicted an increase in stress in C5 of 13%. In contrast, C6 saw an increase in stress with both increases in the nerve root angle (10% and 12% at +6 and +3 degrees) as well as a 23% increase in stress for the decrease in angle of 6 degrees. At a decrease in angle of 3 degrees, the stress in C6 changed only by 1%. Looking at the lower trunk, the predicted stress in the C8 and T1 nerve roots increased for all four changes in angle–up to 67% higher than the control model with a decrease of 6 degrees. The stress at the C7 nerve root increased by between 21% and 30% for both the increases and decreases in the nerve root angle, demonstrating much more consistency compared to other nerve roots.

## Discussion

When it comes to any finite element analysis, limitations exist and must inform the application of the results. In our model, the first limitation stems from the fact there are no neonatal values for mechanical properties of the human infant brachial plexus and spinal cord, thus the needed use of data from neonatal piglets. However, the similarity in structure and size provides a level of confidence that this surrogate is a reasonable match for human tissue of the same age–especially given the range of normal biomechanical properties seen in both species. The second limitation is the inability to model the complete brachial plexus in two dimensions. The need to stop at the division level limits our ability to study the entirety of the brachial plexus and does require loads to be applied through the distal end of the nerve trunk rather than being transmitted from further down the upper extremity.

A 2D model will never fully describe the behavior observed in an actual brachial plexus structure due to the limitations that exist in representing the complex anatomy. However, conducting a stress analysis on an anatomically accurate two-dimensional brachial plexus model allows for validation using available *in vitro* piglet data. Having the ability to validate the 2D model by matching experimentally-determined stress, strain, and force data allows for a level of confidence that this piglet data can be used in the next step, which involves developing a 3D model. Clinical patterns of injury and some experimental work [5] demonstrate that C5 and C6 experience higher stress under initial loading of the brachial plexus than do the lower roots. Our simulation also demonstrated that pattern of stress, as seen in Table 3, even without the complete, complex anatomy of the plexus. This provided an initial level of comfort that the model had some degree of biofidelity. While the applied pressure was distributed based on the nerve root cross-sectional areas, the resulting stress was not constant across the five nerve roots. Thus, the anatomy drove the higher stress at C5 and C6 rather than simply being a balance between applied force and cross-sectional area (Fig 3).

One of the goals of this study was to validate the model against previous literature such that our predicted values of stress were similar to those obtained through experimental testing. As no other model of the human neonatal brachial plexus has to date been published, it is not possible to compare findings from this model to previous models. When the maximum stress predicted at the proximal end of the nerve root from the model was compared to the failure stress determined from testing of piglet brachial plexus mechanical response, the original anatomy was able to reasonably match the stress values at both the high and low deformation rates. While it may be assumed that such a comparison is trivial as both the loading conditions and the material properties came from the same experimental studies, it must be remembered that the anatomy of this 2D model was determined from clinical measurements on humans–not based on the piglet. Thus, while the failure properties of the neonatal piglet brachial plexus (in terms of stress and strain) are expected to be representative of what would be found in a human infant, this model allows for the fact that there are anatomical differences between the species.

When comparing the predicted displacement and strain to the maximum experimental strain, the values predicted by this model were significantly lower than those seen experimentally (Table 5). This is most likely due to the model being developed and analyzed using linear elastic material properties in comparison to a non-linear material. An isotropic linear elastic material can be characterized by two physical constants, including Young’s modulus and Poisson’s ratio. A linear elastic model can be described by a linear relationship between stress and strain–this relationship is known as Hooke’s Law. However, a non-linear elastic material does not obey Hooke’s Law. Biological tissues, including nerves, typically display a region of low stiffness followed by an almost linear increase in the elastic region–ending with a yield stress value that may cause failure to the material or may allow plastic deformation before final rupture occurs. Singh only reported single values for Young’s modulus, and did not include any information on the initial, nonlinear region. However, it is reasonable to assume that the nerve segments did actually demonstrate non-linear behavior. But without any information on that early, low-stiffness behavior of the plexus, the model–using linear elastic properties–is not able to simulate the high deformation-low stress region of the response. Thus, if the non-linear characterization of the neonatal brachial plexus nerve roots is available and can be included in a model, the predicted deformation and strain would be expected to increase–and then more closely match the experimental findings.

**Table 5:** Comparison of predicted displacement and strain compared to maximum experimental strain for Case 1 (low loading-rate properties– 0.01 mm/sec) and Case 2 (high loading-rate properties– 10 mm/sec). Displacement values were identified at the distal end of the trunk.

		Original Segment Length (SC to distal trunk) [mm]	Predicted Displacement [mm]	Maximum Predicted Strain	Experimental Maximum Strain [5]
**Case 1**	**Upper Trunk**	41.4	7.3	17.6%	24%
	**Middle Trunk**	38.3	3.8	9.9%	
	**Lower Trunk**	30.8	4.3	13.9%	
**Case 2**	**Upper Trunk**	41.4	10.6	25.6%	34%
	**Middle Trunk**	38.3	5.3	13.8%	
	**Lower Trunk**	30.8	5.8	18.8%	

As this model’s anatomy only hints at the complex structure seen *in vivo*, it is clear that the results of the analysis can neither be used to predict actual deformation of the nerve roots seen during various loading scenarios nor to assess specific risk of injury for an individual. However, it is appropriate to use the model to investigate specific apples-to-apples comparisons, such as the effect of changes in the angle between the nerve root and the spinal cord.

The examination of the effect of variations in the angle between the nerve root and spinal cord is important because it is well known that there is no single “normal” angle that will be observed among all neonates–anatomical variation is one of the sources of biological variability from one individual to the next. The findings in Phase Two show that a slight variation within the nerve root angles, either an increase or a decrease, significantly changed the stress values at the proximal nerve root, as seen in Table 5. This study provides insight into one of the factors that may affect an individual infant’s susceptibility to brachial plexus injury during the birthing process. It also justifies developing a more complete assessment of the effect of anatomical variations within a 3D model–which is much more complicated.

The implementation of this 2D computational model holds significant implications for clinical practice. First, the computational model offers a unique avenue for exploring mechanisms of NBPP injuries without relying solely on cadaveric studies–by simulating loading scenarios through the model, researchers can gain insight into how various anatomical factors contribute to NBPP risk. As an example, the model allows for the evaluation of anatomical variations in nerve root angles and their impact on stress distribution. This aspect is crucial for understanding individual susceptibility to NBPP during the birthing process. In the future, if imaging allows for detailed visualization of the fetal brachial plexus, clinicians could potentially use such insight to tailor interventions based on an infant’s unique anatomy. In conclusion, by providing a non-invasive and ethically sound method for studying brachial plexus injuries, mechanisms of injuries, and anatomical variations, the developed computational model opens doors for broader research opportunities in the field of biomedical engineering. This is an example of how clinicians and researchers can explore a wider range of scenarios and interventions without ethical concerns associated with traditional cadaveric studies.

In conclusion, this 2D model is the first step towards developing a complete 3D model of the neonatal brachial plexus. Future work will include creating a complex 3D model with all five sections of the brachial plexus–roots, trunks, divisions, cords, and branches. This will provide the opportunity to more accurately assess the effect of the birth process on the stretch within the brachial plexus and the impact of biological variations in structure and properties on the risk of NBPP.
